# The transformation pathways and optimization of conditions for preparation minor ginsenosides from *Panax notoginseng* root by the fungus *Aspergillus tubingensis*

**DOI:** 10.1371/journal.pone.0316279

**Published:** 2025-03-03

**Authors:** Fei-Xing Li, Dong-Mei Lin, Jin Yang, Xiu-Ming Cui, Xiao-Yan Yang

**Affiliations:** 1 Faculty of Life Science and Technology, Kunming University of Science and Technology, Kunming, Yunnan, China; 2 Yunnan Key Laboratory of Sustainable Utilization of Panax Notoginseng, Kunming, Yunnan, China; Foshan University, CHINA

## Abstract

Minor ginsenosides exhibit enhanced pharmacological effects in comparison to the major ginsenosides. However, the natural content of minor ginsenosides in plants is typically insufficient to satisfy clinical demand. Therefore, we investigated the biotransformation of the major ginsenosides in *Panax notoginseng* to minor ginsenosides by the fungus *Aspergillus tubingensis*. The transformation products were analyzed using TLC, HPLC, and LC-MS techniques to propose the biotransformation pathways of major ginsenosides. *A. tubingensis* was found to transform the main ginsenosides into 15 minor ginsenosides, inculding (*R*/*S*)-Rg_*3*_, Rk_1_, Rg_5_, F_2_, (*R*/*S*)-Rh_1_, Rk_3_, Rh_4_, (*R*/*S*)-Rg_2_, F_4_, Rg_6_ and (*R*/*S*)-R_2_. The transformation reactions encompassed isomerization, hydrolysis and dehydration. We have also optimized the reaction temperature and pH for the crude enzyme extracted from this fungus, which has a molecular weight of 66 kDa. Based on our current knowledge, this transformative characteristic of *A. tubingensis* was initially documented for the concurrent transformation of PPD and PPT type saponins in *P. notoginseng*. This method of preparing minor saponins will be valuable for the development of *P. notoginseng* as a traditional medicinal material.

## 1. Introduction

*Panax notoginseng* (Burk.) F. H. Chen is a highly famous traditional Chinese medicinal materials. Its medicinal parts are the dried roots and rhizomes, and the main bioactive ingredients of *P. notoginseng* are saponins, which exhibit a variety of pharmacological activities [1–2]. The primary ingredient of *P. notoginseng* root are protopanaxadiol (PPD)-type ginsenosides and protopanaxatriol (PPT)-type ginsenosides [3–4]. Among these saponins, the ginsenosides Rb_1_, Rd, Re and Rg_1_, as well as the notoginsenoside R_1_, account for over 80% of the total saponins [5], and they are therefore known as major ginsenosides [6]. These major ginsenosides tend to be greater polarity due to the presence of more sugar groups at positions C-3, C-6 and C-20 of the ginsensides. Because of this polarity, orally administered saponins are not readily absorbed in the small intestine and thus are characterized by low bioavailability. Therefore, to enhance the bioavailability of these saponins, it is necessary to remove glucopyranosyl at C-3, C-6 and C-20 through hydrolysis to generate minor ginsenosides with fewer glycosyl groups. As expected, the pharmacological activities of these minor ginsenosides, including Rg_3_, Rh_1_, and Rh_2_, have been shown to be higher than those of the main naturally occurring ginsenosides [7–9]. In particular, the minor ginsenosides Rg_3_, Rg_2_, Rh_4_, C-K and F_2_ have been found to exert anti-cancer activity [10–14], and minor ginsenosides Rh_2_, Rk_1_, Rk_3_, Rg_5_ and Rg_6_ exert anti-tumor activity [15–19].

Obtaining significant amounts of minor ginsenosides is challenging by isolation from *P. notoginseng* root because of their low abundance. Due to the fact that minor ginsenosides and major ginsenosides share the same parent nucleus in their structures, so they can be derived from major saponins. One key way to obtain these minor ginsenosides involves biological transformation, which relies on microbial strains or enzymes produced by them to hydrolyze, dehydrate, oxidize and isomerize the substrate saponins, so as to obtain minor saponins with high pharmacological activity. Numerous studies have investigated the biological transformation of major saponins into minor ginsenosides. As an illustration, ginsenoside Rb_1_ can be transformed to Rd, F_2_ and C-K by *Aspergillus niger* [20]. In addition, the PPD-type saponins were found to be transformed into C-K with higher pharmacological activity, by *A. tubingensis* isolated from fermented soybean [21]. Ginsenoside Rb_2_ can be transformed to C-O, C-Y and C-K by ginsenosidase type-I from *A. niger* g.848 [22], and ginsenosides Rb_1_, Rb_2_, Rc, Rd and Rg_3_ were found to be completely transformed into Gyp-XVII, C-O, C-Mc_1_, F_2_ and Rh_2_, respectively, by *A. oryzae* [23]. Overall, due to its efficient product generation, mild reaction conditions and high conversion rate, biological transformation represents an important tool in ginsenoside transformation [24,25].

In this study, the properties of the biological transformations of ginsenosides Rb_1_, Rd, Re and Rg_1_ and notoginsenoside R_1_ by *A. tubingensis* were studied. The transformation products of major ginsenosides were analyzed using chromatographic and mass spectrometric methods, leading to the proposal of biotransformation pathways for major ginsenosides. Furthermore, the enzyme responsible for these transformations, *β*-ginsenosidase, was purified and characterized. We found that the enzyme isolated from *A. tubingensis* was over a wide temperature range and the enzyme activity remained above 70% which formed the basis for its large-scale preparation.

## 2. Materials and methods

### 2.1. Materials

The standard ginsenosides Rb_1_, Rd, R_1_, Rg_1_, Re, (*R*/*S*)-R_2_, (*R*/*S*)-Rg_2_, (*R*/*S*)-Rh_1_, (*R*/*S*)-Rg_3_, T_5_, Rg_6_, F_4_, Rk_3_, Rh_4_, F_2_, CK, Rk_1_ and Rg_5_ were purchased from Vicky Biotechnology Co., Ltd., China. DEAE-52 was purchased from Shanghai Yuan ye Biological Co., Ltd., China. The BCA protein concentration assay kit was purchased from Beyotime Biotechnology, China. *A. tubingensis* was isolated from soil in which *P. notoginseng* was growing. The standard proteins were purchased from Takara Bio, Inc. (Otsu, Shiga, Japan). Column chromatography (CC) was performed using a macroporous resin D101 (‌Shanghai Yuanye Bio-Technology Co., Ltd, China), silica gel (200-300 mesh; Qingdao Marine Chemical Ltd., China), RP-18 gel (40-75 μm, Fuji Silysia Chemical Ltd., Japan), and Sephadex LH-20 (Amersham Biosciences, Sweden). Fractions were monitored by TLC (GF 254, Qingdao Haiyang Chemical Co., Ltd. Qingdao).

*P. notoginseng* was collected from Wenshan County, Yunnan Province, China, in August 2022, and identified by Prof Xiuming Cui, Kunming University of Science and Technology. The voucher specimen is No. YXY20220820. The sample collection site is our school’s experimental research base. All of our research has been approved by the Kunming University of Science and Technology.

### 2.2. Isolation and identification of fungus

*A. tubingensis* was isolated from the cultivation soil of *P. notoginseng* in the Laboratory Greenhouse for *P. notoginseng* cultivation, Kunming University of Science and Technology, China. The RSA login number of the strain was recorded with NCBI as SRR2285937. Morphological characteristics of *A. tubingensis* and its spores were observed visually and recorded, and the spore morphology was observed by microscopy. The Kunming Branch of Tsingke Biotechnology Co., Ltd. conducted the amplification and sequencing of the internal transcribed spacer (ITS) rDNA gene. The sequencing results were uploaded to NCBI for comparison. Utilizing MEGA 7.0, a Neighbor-Joining (NJ) phylogenetic tree was constructed by selecting species exhibiting significant homology.

### 2.3. Isolation and purification of five monomeric saponins from total saponins of *P. notoginseng* root

After crushing the main root of *P. notoginseng* (200 g), the main saponins were extracted by ethanol reflux extraction [26]. This extraction process utilized a 75% ethanol solution and a solid-liquid ratio of 1:10. After extraction, solid-liquid separation was carried out, and the separated liquid was extracted by adding 2 times volume of water-saturated n-butanol. The upper liquid was used for vacuum evaporation and concentration to obtain the crude extract of total saponins (50 g). The crude extract of total saponins were preliminarily purified by macroporous resin D101 column chromatography (CC), eluted with EtOH-H_2_O (60-100%, v/v), to afford five fractions (Frs. A-E). Fr. B was further eluted with CH_2_Cl_2_ - MeOH (10:3 to 10:5, v/v) over silica gel CC to obtain Frs. B_1_-B_4_. Among them, Frs. B_1_, B_2_, and B_4_ were further purified by Sephadex LH-20 CC eluting with MeOH and Rp-18 silica gel CC eluting with MeOH-H_2_O (20-80%) to obtain compounds **1** (173 mg), **2** (87 mg), and **5** (158 mg). Fr. B_3_ was subjected to a MPLC with a stepwise gradient of MeOH-H_2_O (60-100%) to afford compounds **3** (124 mg) and **4** (103 mg).

### 2.4. Biotransformation of monomer ginsenosides Rb_1_, Rd, Re, Rg_1_ and notoginsenoside R_1_ by *A. tubingensis*

Biotransformation experiments were performed by adding ginsenosides to potato dextrose broth (PDB) liquid medium containing the fungus. Control experiments were performed in medium with only fungus. The biotransformation experiments were performed using PDB medium containing monomeric ginsenoside. The samples were incubated in a shaking incubator (160 rpm) at 26 °C for 21 days, respectively. The yield can be calculated using the following formula:


$$\text{Yield rate }\left(\text{\%}\right)=\frac{\text{amount of transformed product}}{\text{amount of transformed substrate}}\times 100\text{\%}$$

### 2.5. Preparation and preliminary purification of crude enzyme from *A. tubingensis*

To prepare a strain seed liquid, *A. tubingensis* was inoculated into PDB medium, and the mixture was cultured at 26 °C and 160 rpm for 5 days. To ensure consistent strain growth rate in subsequent cultures, equal amounts (10 mL) of seed fluid were added to 600 mL of PDB medium and cultured for 7 days at 26°C and 160 rpm. Culture medium: potato extract powder (5.0 g/L), glucose (15.0 g/L), pH natural. After the fermentation solution was collected, (NH_4_)_2_SO_4_ was added to the fermentation solution for protein precipitation. The addition amount of (NH_4_)_2_SO_4_ was: 80g (NH_4_)_2_SO_4_ was added to 100mL fermentation solution. The protein solution was dialyzed extensively and then freeze-dried according to a previously described method [27].

The enzyme was purified over a DEAE-cellulose DE-52 column (*ϕ*2 cm ×  20 cm, Whatman). The enzymatic activity of fractions from this purification step were assayed using the substrate ginsenoside Rb_1_. Ginsenoside Rb_1_ hydrolysis was assessed via TLC, followed by collection and lyophilization of a portion of the hydrolyzed compound.

The crude enzyme that has been preliminarily purified was analyzed by polyacrylamide gel electrophoresis and determined by BCA protein concentration assay kit [28]. The crude enzyme, having undergone initial purification, underwent assay to determine its specific activity. The p-Nitrophenyl-*β*-D-glucopyranoside (pNPG) served as the substrate. The activity of *β*-glucosidase was quantified, defining one unit as the enzyme quantity yielding 1 μmol of p-nitrophenol (pNP) per minute in a 1 mL enzyme solution [29].

### 2.6. Crude enzyme basic characterization

#### 2.6.1 Effect of pH on the activity and stability of *β*‑glucosidase.

The impact of pH on *β*-glucosidase activity was evaluated by utilizing pNPG as the substrate. The rates of reaction of the enzyme in sodium acetate buffers with pH values in the range of 4 to 8 were respectively determined following incubation at 50 °C for 20 min. The crude enzyme solution (30 mg/mL) was thoroughly mixed with a solution volume ratio of 2:1 for pNPG (1 mM) and incubated at 50°C for 20 min, followed by the addition of 0.5 M NaOH to halt the reaction and absorbances were read at 405 nm. The pH stability of the enzyme was assessed by incubating the *β*-glucosidase buffers with pH values from 4 to 8 for 0, 10, 20, 30, 40, and 50 min at 50 °C. The activities of *β*-glucosidase were measured by a colourimetric method using pNPG as substrate. Maximum activities were expressed relative to the maximum enzyme activity in this series of experiments. All tests were performed in triplicate.

#### 2.6.2 Effect of temperature on the activity and stability of *β*‑glucosidase.

The effects of temperature on *β*-glucosidase activity was evaluated using pNPG as the substrate. For optimal temperature testing, the reaction mixture was determined in NaC_2_H_3_O_2_ buffer (pH 5.0) a range of temperatures from 30 to 70 °C for 20 min. The crude enzyme solution (30 mg/mL) was thoroughly mixed with a solution volume ratio of 2:1 for pNPG (1 mM) and incubated at 50°C for 20 min, followed by the addition of 0.5 M NaOH to halt the reaction and absorbances were read at 405 nm. The enzyme’s temperature stability was evaluated by incubating the *β*-glucosidase in the NaC_2_H_3_O_2_ buffer (pH 5.0) for 0, 10, 20, 30, 40, and 50 min at 30, 40, 50, 55, 60 or 70°C. The activities of *β*-glucosidase were measured by a colourimetric method using pNPG as substrate. Maximum activities were expressed relative to the maximum enzyme activity in this series of experiments. All tests were performed in triplicate.

### 2.7. Analytical methods

The thin layer chromatography (TLC) was performed using HSGF_254_ silica gel plates with chloroform: methanol: water (2:1:0.1, v/v/v) as the developing solvent. Spots on the TLC plates were identified by staining with 10% (v/v) H_2_SO_4_ in ethanol and heating at 110 °C for 2 min.

The identification of transformation products was achieved by liquid chromatography using an Agilent system (Grand Island, NY, USA) with an ACQUITY UPLC BEH C_18_ column (1.7 µm, 2.1 × 50 mm, Waters, USA). The column was subjected to a gradient elution using water as mobile phase A and acetonitrile as mobile phase B. The elution gradient was programmed as follows: 0-3 min, 20% B; 3-5 min, B from 20% to 30%; 5-6 min, B from 30% to 35%; 6-8 min, B from 35% to 40%; 8-16 min, 40% B; 16-30 min, B form 40% to 45%; 30-45 min, B form 45% to 75%; and 45-55 min, B form 75% to 95%. The flow rate was 0.3 mL/min, samples were detected by absorption at 203 nm with an injection volume of 10 µ L, and the column temperature was maintained at 30 °C.

## 3. Results and discussion

### 3.1. Characterization of *A. tubingensis*

**Fig 1:**
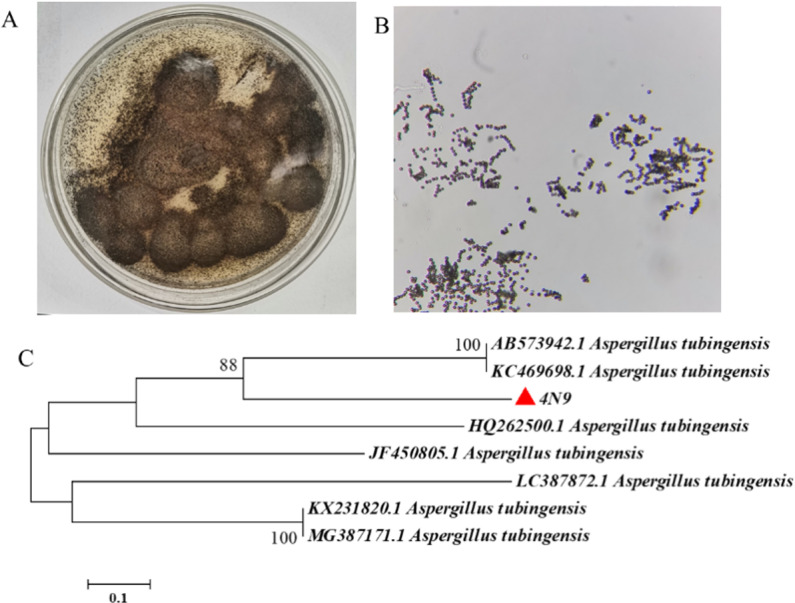
Morphology and phylogenetic tree of *A. tubingensis.* (A) Colony morphology diagram. (B) Conidiophore of *A. tubingensis*. (C) The phylogenetic tree based on ITS rDNA gene sequences of *A. tubingensis*.

We isolated a fungus from the soil in which *P. notoginseng* was growing, and we predicted that this fungus would be *A. tubingensis*. The ensuing colonies were found to be unevenly distributed throughout the plate, granular, and black in color (Fig 1A). When observed under a microscope, the cells were seen to have formed dark-brown to black conidia (Fig 1B). These features are consistent with those described in other studies of *A. tubingensis* [30,31]. When the sequence of the ITS rDNA gene of the strain was determined (supplemental file) and compared to sequences in the GenBank database, and this strain was identified as being in the genus *Aspergillus*, which sequence indicated a great deal of resemblance to the ITS rDNA gene from several strains of *A. tubingensis* (Fig 1C).

### 3.2. Isolation and purification of main saponins from *P. notoginseng* root

Isolation and purification of five main saponins compounds **1**–**5** from *P. notoginseng* root. Compounds **1**–**5** were identified as ginsenoside Rg_1_ (**1**), notoginsenoside R_1_ (**2**), ginsenoside Re (**3**), ginsenoside Rd (**4**) and ginsenoside Rb_1_ (**5**), respectively. Their structures were elucidated by the comparison of their ^13^C NMR data (S1 Table) with those of literature [32–34].

### 3.3. Biotransformation of major ginsenosides of *P. notoginseng* root by *A. tubingensis*

**Fig 2:**
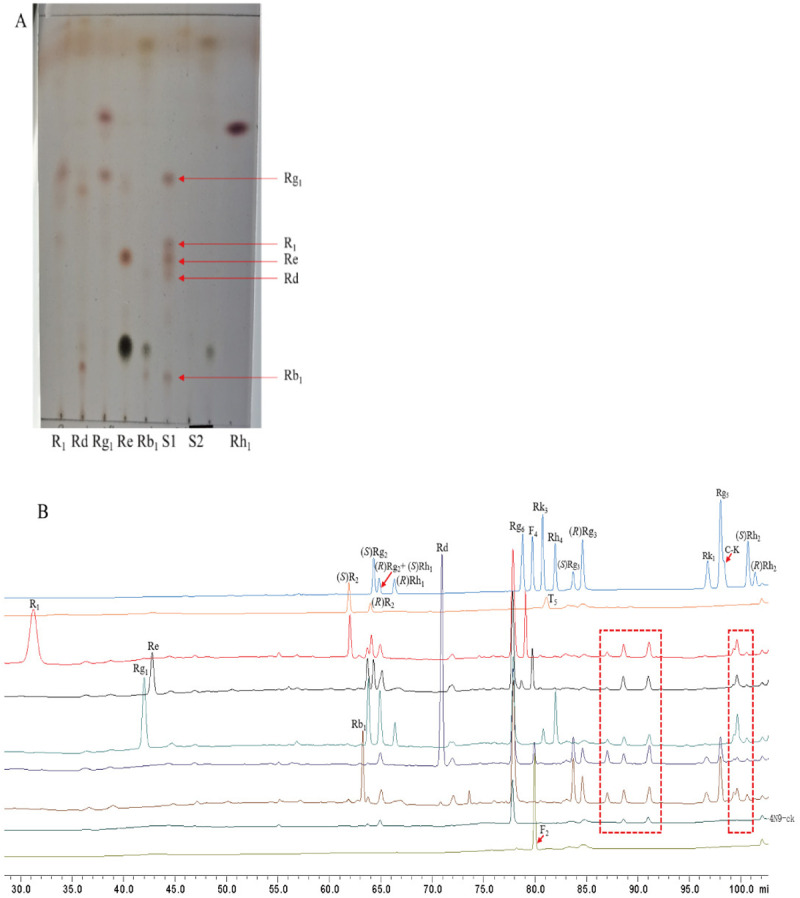
Analyses of the transformation products of five monomer ginsenosides by *A. tubingensis.* (A) TLC analysis of the transformation products by *A. tubingensis*. Rb_1_, Rd, Re, Rg_1_ and R_1_: Representing their respective transformed products; S1: Authentic ginsenosides mixtures; S2: Metabolites of the *A. tubingensis*; (B) HPLC analysis of the transformation products by *A. tubingensis*. Rb_1_, Rd, Re, Rg_1_ and R_1_: Representing their respective transformed products; 4N9-ck: Metabolites of the *A. tubingensis* (control, without added substrate); The red dashed boxes represent strain metabolites or unknown saponins.

Major ginsenosides were subjected to biotransformation by *A. tubingensis*. The outcomes demonstrated *A. tubingensis* possesses a remarkable capacity to transformed the major ginsenosides. The presence of polar spots on TLC that are smaller than the polarity of the substrate suggests that minor saponins are forming (Fig 2A). Similarly, the results of the HPLC (Fig 2B) also indicated the production of several minor saponins.

**Fig 3:**
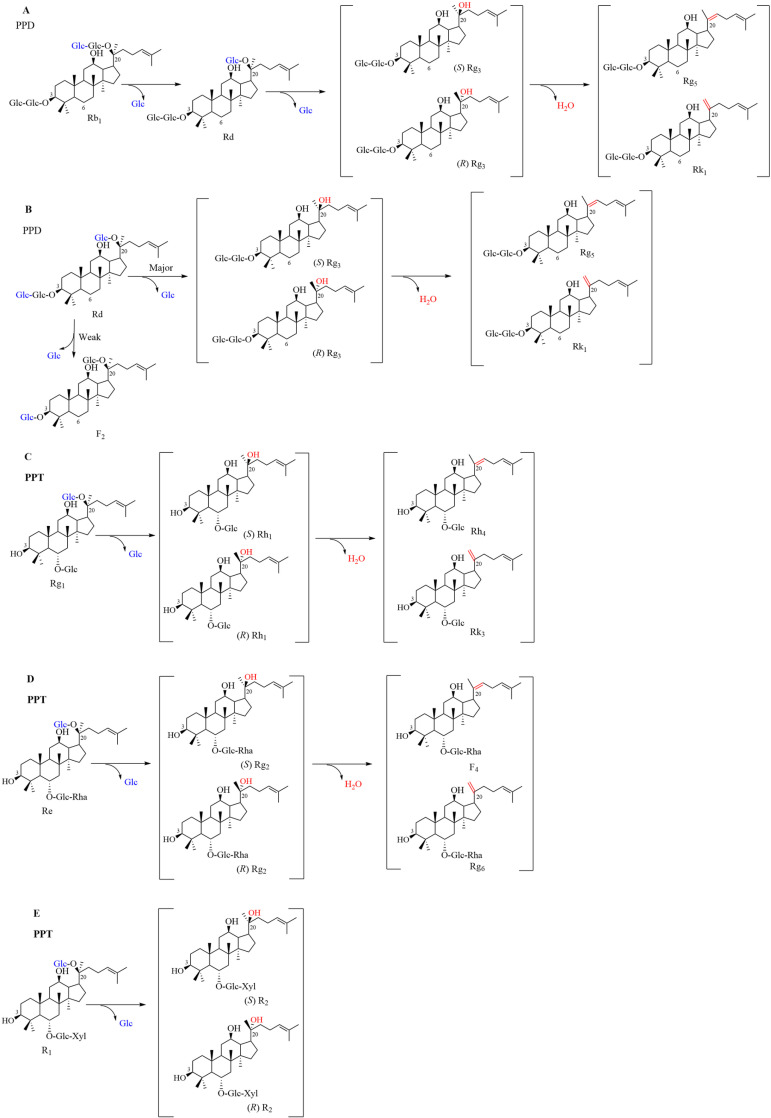
The proposed biotransformation pathways of five ginsenosides by *A. tubingensis.* The proposed biotransformation pathways (A-E) of ginsenosides Rb_1_, Rd, Rg_1_, Re and notoginsenoside R_1_ by *A. tubingensis*.

Based on the chromatography findings, we proposed biotransformation pathways for the primary ginsenosides in *P. notoginseng* root. Each resultant product underwent additional scrutiny using LC-MS analysis. The Fig 3A depicts the proposed transformation pathway of ginsenoside Rb_1_. The HPLC chromatogram (Fig 2B) revealed peaks attributed to small polar products, and these compounds were identified as Rd, (*R/S*)-Rg_3_, Rk_1_ and Rg_5_. Accordingly, we suggest that Rb_1_ was biotransformed into Rd, (*R/S*)-Rg_3_, Rk_1_ and Rg_5_ by *A. tubingensis.* In addition, the results can also be illustrated in LC-MS analysis (S1A Fig).

Based on the established attributes of saponin transformation routes, which typically hydrolyze single glycosyl fragments, we proposed that an enzyme from *A. tubingensis* hydrolyzed the 20-O-*β*-D-(1 → 6)-glucopyranoside bond of Rb_1_, yielding Rd. Subsequently, it catalyzed the hydrolysis of the inner 20-O-*β*-D-glucopyranoside moiety of Rd, resulting in the formation of (*R*/*S*)-Rg_3_. Finally, it facilitated the dehydration process of the tertiary hydroxy group in (*R*/*S*)-Rg_3_, generating Rk_1_ and Rg_5_. Therefore, the following was the transformation pathway of ginsenoside Rb_1_: Rb_1_ → Rd → (*R/S*)-Rg_3_ → Rk_1_ and Rg_5_.

The proposed pathway of transformation of ginsenoside Rd is displayed in Fig 3B. Regarding the outcomes of the HPLC analysis (Fig 2B), we concluded that small polar compounds were present in the product, and these molecules were identified as F_2_, (*R/S*)-Rg_3_, Rk_1_ and Rg_5_. Therefore, we suggest that Rd was biotransformed into F_2_, (*R/S*)-Rg_3_, Rk_1_ and Rg_5_ by *A. tubingensis.* Similarly, the results can also be illustrated in LC-MS analysis (S1B Fig).

The enzyme from *A. tubingensis* can hydrolyzed the ginsenoside Rd to F_2_ or Rd to (*R*/*S*)-Rg_3_, and finally removed the tertiary hydroxy group in (*R/S*)-Rg_3_ via dehydration to form Rk_1_ and Rg_5_. Therefore, the transformation routes of ginsenoside Rd were as follows: Rd → (*R/S*)-Rg_3_ → Rk_1_ and Rg_5_, and Rd → F_2_.

The Fig 3C shows the presumed transformation process of ginsenoside Rg_1_. The small polar compounds present in the product as shown by HPLC analysis (Fig 2B) identified as (*R/S*)-Rh_1_, Rk_3_ and Rh_4_. We suggest, then, that Rg_1_ was biotransformed into (*R/S*)-Rh_1_, Rk_3_ and Rh_4_ by *A. tubingensis.* Ultimately, the transformed outcomes of ginsenoside Rg_1_ were evaluated by LC-MS (S1C Fig). According to the characteristics of saponin transformation pathways, we propose that the enzyme from *A. tubingensis* hydrolyzed the 20-O-*β*-D-glucopyranoside bond of Rg_1_ to form Rh_1_ and then removed the tertiary hydroxy group in (*R/S*)-Rh_1_ via a dehydration reaction to form Rk_3_ and Rh_4_. Therefore, the transformation pathway of ginsenoside Rb_1_ was proposed to be Rg_1_ → (*R/S*)-Rh_1_ → Rk_3_ and Rh_4_. Similarly, the transformation pathway of ginsenoside Re and notoginsenoside R_1_ were proposed to be Re → (*R/S*)-Rg_2_ → F_4_ and Rg_6_ (Fig 3D and S1D Figs) and R_1_ → (*R/S*)-R_2_ (Fig 3E and S1E Figs), respectively.

In HPLC analyses of the transformation pathways of ginsenosides Rb_1_ and Rd, the peaks corresponding to the products of transformation of ginsenoside Rd were consistent with the retention time of standard ginsenoside F_2_, while no peaks coeluting with standard F_2_ were seen when analyzing the products of transformation of ginsenoside Rb_1_. According to the hydrolysis pathways observed for other saponins, we predicted that ginsenoside F_2_ would be hydrolyzed to C-K and that ginsenoside C-K would be hydrolyzed to Rh_2_, but we did not observe C-K or Rh_2_ in the transformation products; accordingly, we believe that the pathway of Rd hydrolysis to F_2_ is relatively minor. The yield (%) of the minor ginsenosides from the major ginsenoside in *P. notoginseng* root upon transformation by *A. tubingensis* are shown in Table 1.

**Table 1:** The yields of the minor ginsenosides upon biotransformation of major ginsenosides from *P. notoginseng* root by *A. tubingensis.*

Substrates		Products	Yield (%)
PPD	Rb_1_	(*R/S*)-Rg_3_, Rk_1_, Rg_5_	23, 18, 8, 24
	Rd	F_2_, (*R/S*)-Rg_3_, Rk_1_, Rg_5_	5, 8, 7, 4, 9
PPT	Rg_1_	(*R/S*)-Rh_1_, Rk_3_, Rh_4_	19, 7, 4, 14
	Re	(*R/S*)-Rg_2_, F_4_, Rg_6_	20, 17, 9, 23
	R_1_	*(R/S)-*R_2_	7, 12

### 3.4. Purification and characterization of the biotransformation enzyme from *A. tubingensis*

In order to identify the enzyme responsible for the biotransformation, the supernatant was processed by anion exchange chromatography. Following the addition of the crude enzyme to the packed column, 0.05 M to 1.0 M KCl was progressively added to the column for elution. Eluted proteins were collected in 5.0 mL fractions.

Partial solutions of each fraction were mixed with ginsenoside Rb_1_ and incubated for 48 h at 50 °C to measure the level of enzyme activity in each fraction. TLC is used to detect the transformation effect. Fractions 130 through 133, which contained proteins eluted with 0.4 M KCl, were found to hydrolyze ginsenoside Rb_1_ into another ginsenoside with the highest activity (S2A Fig). When performing gel electrophoresis analysis, the molecular weight of the crude enzyme protein after preliminary purification is about 66 kDa (S2B Fig). The results of this crude enzyme protein is similar to those reported previously in the literature [4,35]. Considering the entire purification, the yield of enzyme was 3.4%, and the specific activity was increased by 4.8-fold (Table 2).

**Table 2:** Purification of *β*-glucosidase from *A. tubingensis.*

Step	Total protein (mg)	Total units (U)	Specific activity (U/mg)	Purification (-fold)	Yield (%)
Culture liquid	1534.0	21150.0	13.8	1	100
Precipitation of (NH_4_)_2_SO_4_	65.28	2014.0	30.9	2.3	9.5
DEAE-cellulose	14.9	723.0	62.0	4.8	3.4

### 3.5. Effects of pH and temperature on the activity and stability of *β*-glucosidase from *A. tubingensis*

**Fig 4:**
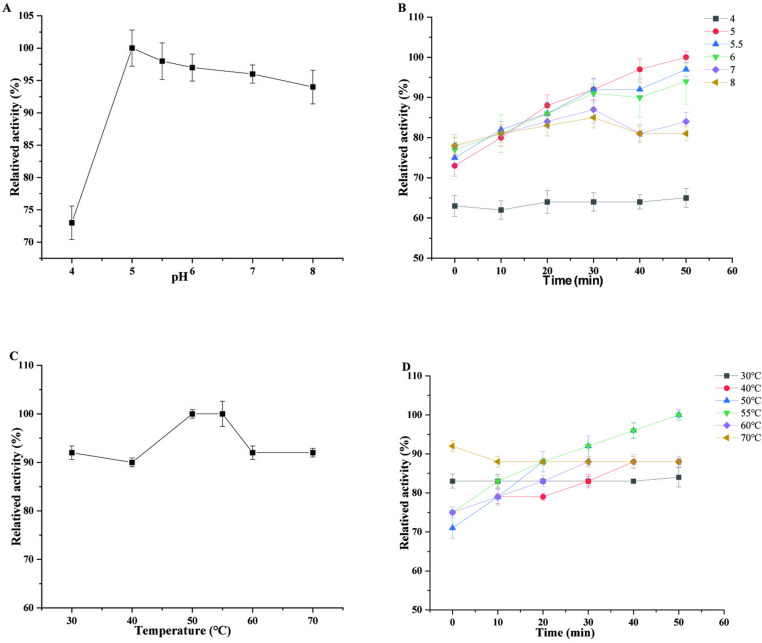
Effects of pH and temperature on the activity and stability of ***β*-glucosidase from *A. tubingensis***. (A) The effect of pH on the activity of *β*-glucosidase. (B) The effect of pH on the stability of *β*-glucosidase. (C) The effect of temperature on the activity of *β*-glucosidase. (D) The effect of temperature on the stability of *β*-glucosidase.

The Fig 4 illustrates the influence of temperature and pH on enzyme activity. The optimal pH of enzyme from *A. tubingensis* was pH 5, which was a relatively high activity (Fig 4A). The enzyme from *A. tubingensis* was found to be stable at a relatively wide range of pH values. As shown in Fig 4B, the enzyme from *A. tubingensis* was most stable in buffers with a pH range of 5 to 8, retaining approximately 70% of its activity. While the enzyme retained activity when incubated at pH 4, the enzyme activity was lower as compared to incubation at other pH levels.

The temperature at which the enzyme from *A. tubingensis* has the highest activity was found to be 50 and 55 °C (Fig 4C). Furthermore, the enzyme from *A. tubingensis* was also found to be stable at relatively high temperatures. As it shown in Fig 4D, when the enzyme was incubated at different temperatures and then assayed for activity, it was found to be particularly stable over the range of 30 °C to 70 °C, and the enzyme activity was maintained at approximately 70%. It has been reported that the optimal catalytic temperature of *β*-glucosidase from animals and plants is approximately 40°C, while the optimal catalytic temperature of *β*-glucosidase from microorganisms is higher, and the microorganismal enzyme is generally stable below 60 °C [36]. In this study, the enzyme from *A. tubingensis* retained activity at 70 °C, which implies that the enzyme is flexible with respect to temperature.

## 4. Conclusion

To improve the utilization of *P. notoginseng*, the PPD and PPT ginsenosides from *P. notoginseng* root were converted by the *A. tubingensis* to prepare minor ginsenosides. In this study, *A. tubingensis* was found to has the capable of convert major ginsenosides in *P. notoginseng* root (Rb_1_, Rg_1_, Re, and Rd and notoginsenoside R_1_) into 15 minor ginsenosides. Besides, according to HPLC analyses of the transformation products of *P. notoginseng* ginsenosides converted by *A. tubingensis*, we found that six unknown compounds appeared during the conversion process. If we expand the scale of transformation, we may identify unknown components in the transformation products through isolation and identification methods. Therefore, it is possible to identify more minor ginsenosides and even new ginsenoside derivatives from the saponin conversion products.

The fungus can efficiently and selectively hydrolyze major ginsenosides in *P. notoginseng* root into minor ginsenosides involves three main types of reactions: hydrolysis, epimerization, and dehydration. In PPD-type ginsenosides, the major reaction involves the hydrolysis of the glucosyl moiety linked to C-20 and C-3. In PPT-type ginsenosides, we did not find evidence that the transformation reaction involves hydrolysis of the glucosyl moiety attached to C-6. Both types of saponins undergo isomerization and dehydration during the conversion process, resulting in the formation of 20(*S,R*)-epimers and double-bond isomers. Different from other *Aspergillus* species, it showed that *A. tubingensis* was able to form C-20(22) double-bond isomers by dehydration. For example, though the conversion reactions, the ginsenosides Rk_1_, Rk_3_, Rg_5_ and Rg_6_ can be formed, which are known to have antitumor activity.

The saponin transformation activity of *A. tubingensis* is notably robust, enabling it to concurrently convert both PPD and PPT ginsenosides from *P. notoginseng*. The resulting transformation products are quite diverse. Through meticulous analysis, we identified 15 minor ginsenosides among these transformation products. Furthermore, we have proposed the transformation pathways of ginsenosides, offering a robust theoretical foundation for the preparation and acquisition of minor ginsenosides. Our study provides a solid theoretical foundation and practical methods for the large-scale production of minor ginsenosides.

## Supporting information

S1 Text The sequencing of the ITS rDNA gene of *Aspergillus tubingensis.
* (DOCX)

S1 Fig LC-MS analysis of transformation products of ginsenosides Rb_1_, Rd, Rg_1_, Re, notoginsenoside R_1_ by *A. tubingensis.
* (TIFF)

S2 Fig Enzyme characterization of *β*-ginsenosidase. (A) TLC analysis of ginsenosides Rb1 transformed by enzyme of different fractions. C: authentic ginsenosides; S1-S2: the fraction enzyme of ginsenoside-transformation activity; S3-5: the other fraction enzyme. (B) SDS-PAGE analysis of the purified *β*-glucosidase from *A*. tubingensis after protein staining with Coomassie Brilliant Blue solution. M: protein marker, a-f: purified enzyme. (TIFF)

S3 Fig ^13^C spectrum (150MHz, C_5_D_5_N) of 1. (TIF)

S4 Fig ^13^C spectrum (150MHz, C_5_D_5_N) of 2. (TIF)

S5 Fig ^13^C spectrum (150MHz, C_5_D_5_N) of 3. (TIF)

S6 Fig ^13^C spectrum (150MHz, C_5_D_5_N) of 4. (TIF)

S7 Fig ^13^C spectrum (150MHz, C_5_D_5_N) of 5. (TIF)

S1 Table ^3^C NMR data for compounds 1–5 in C_5_D_5_N. (DOCX)
