# Supplementary material for: The transformation pathways and optimization of conditions for preparation minor ginsenosides from Panax notoginseng root by the fungus Aspergillus tubingensis
Source: PLoS One. 2025 Mar 3;20(3):e0316279. doi: 10.1371/journal.pone.0316279 (PMC11875379; doi:10.1371/journal.pone.0316279)
Supplement: S1 Text — (DOCX) [file pone.0316279.s001.docx]

**Supplemental Data**

**The transformation pathways and optimization of conditions for preparation minor ginsenosides from *Panax notoginseng* root by the fungus *Aspergillus tubingensis***

Fei-Xing Li^1☯^, Dong-Mei Lin^1☯^, Jin Yang^1^, Xiu-Ming Cui^1^, Xiao-Yan Yang^1,2*^

^1^Faculty of Life Science and Technology, Kunming University of Science and Technology, Kunming, Yunnan, China;

^2^Yunnan Key Laboratory of Sustainable Utilization of *Panax Notoginseng*, Kunming, Yunnan, China

^☯^These authors contributed equally to this work.

* Corresponding author

E-mail: [yangxy@kust.edu.cn](mailto:yangxy@kust.edu.cn)

**S1 Text.** The sequencing of the ITS rDNA gene of *A. tubingensis*. --------------------------------------3

**S1 Fig.** LC-MS analysis of transformation products of ginsenosides Rb_1_, Rd, Rg_1_, Re, notoginsenoside R_1_ by *A.tubingensis*. ---------------------------------------------------------------------4-8

**S2 Fig.** Enzyme characterization of *β*-ginsenosidase. -----------------------------------------------------9

**S3 Fig.** ^13^C spectrum (150MHz, C_5_D_5_N) of **1**. -------------------------------------------------------------10

**S4 Fig.** ^13^C spectrum (150MHz, C_5_D_5_N) of **2**. -------------------------------------------------------------10

**S5 Fig.** ^13^C spectrum (150MHz, C_5_D_5_N) of **3**. -------------------------------------------------------------11

**S6 Fig.** ^13^C spectrum (150MHz, C_5_D_5_N) of **4**. -------------------------------------------------------------11

**S7 Fig.** ^13^C spectrum (150MHz, C_5_D_5_N) of **5**. -------------------------------------------------------------12

**S1 Table.** ^13^C NMR data for compounds **1**–**5** in C_5_D_5_N. ------------------------------------------------13

**S1 Text.** The sequencing of the ITS rDNA gene of *A. tubingensis*.

ATGGGAGCTACCTGATCCGAGGTCACCTGGAAAAAATGGTTGGAAAACGTCGGCAGGCGCCGGCCAATCCTACAGAGCATGTGACAAAGCCCCATACGCTCGAGGATCGGACGCGGTGCCGCCGCTGCCTTTCGGGCCCGTCCCCCCGGAGAGGGGGACGGCGACCCAACACACAAGCCGGGCTTGAGGGCAGCAATGACGCTCGGACAGGCATGCCCCCCGGAATACCAGGGGGCGCAATGTGCGTTCAAAGACTCGATGATTCACTGAATTCTGCAATTCACATTAGTTATCGCATTTCGCTGCGTTCTTCATCGATGCCGGAACCAAGAGATCCATTGTTGAAAGTTTTAACTGATTGCATTCAATCAACTCAGACTGCACGCTTTCAGACAGTGTTCGTGTTGGGGTCTCCGGCGGGCACGGGCCCGGGGGGCAAAGGCGCCCCCCCGGCGGCCGACAAGCGGCGGGCCCGCCGAAGCAACAGGGTATAATAGACACGGATGGGAGGTTGGGCCCAAAGGACCCGCACTCGGTAATGATCCTTCCGCAGGTTCCCCTAACGAGAAGT

A


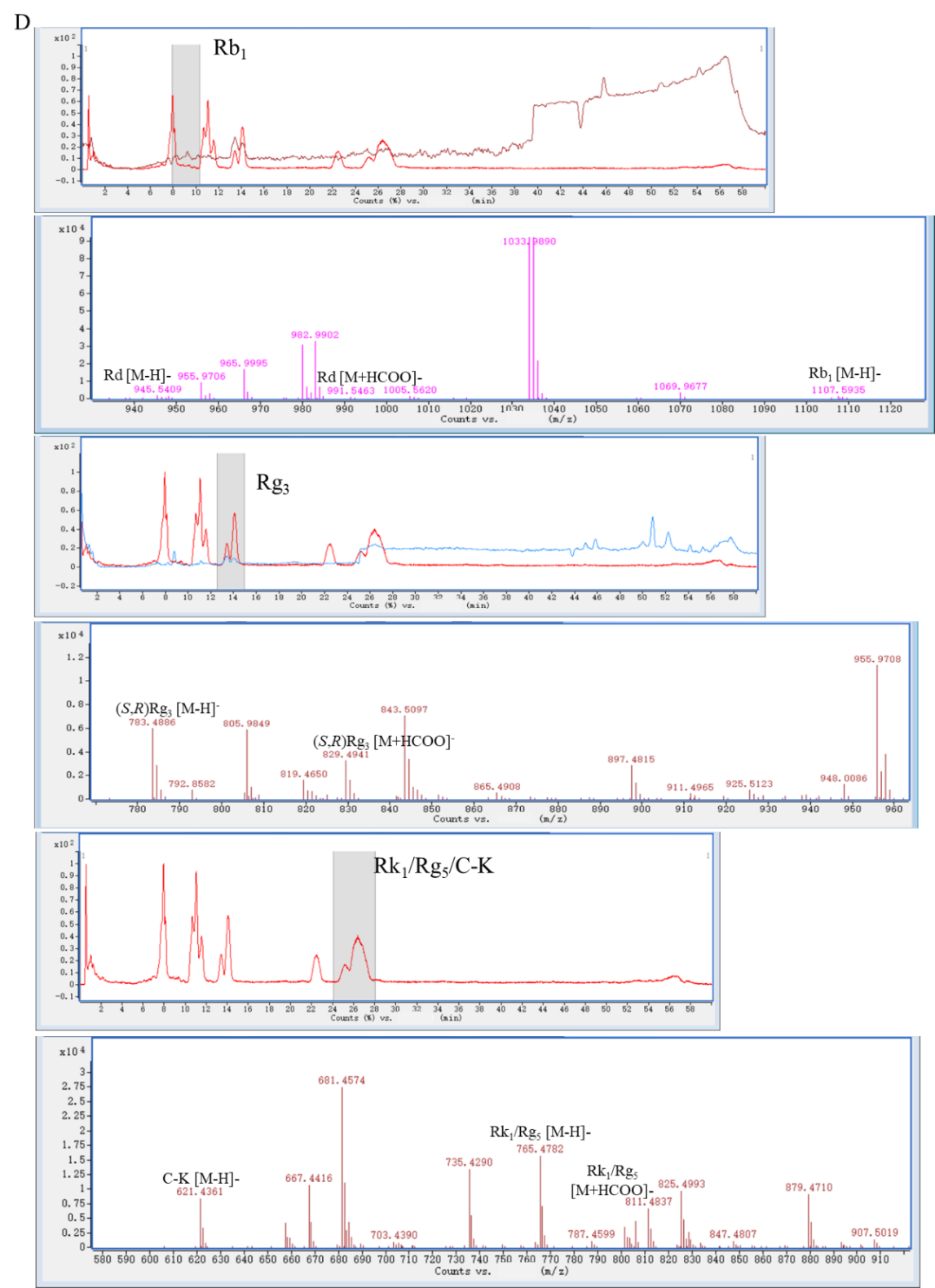


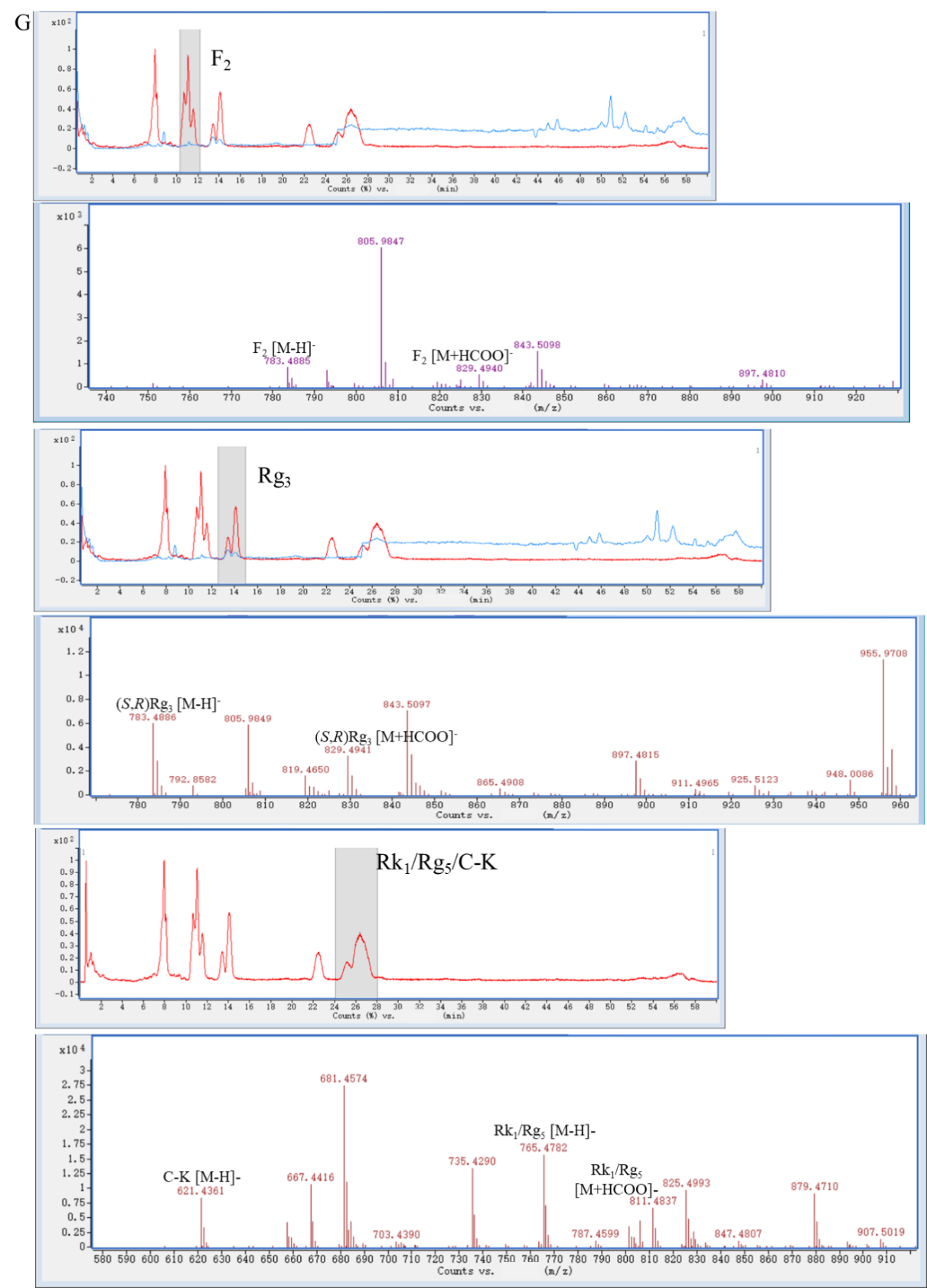


B


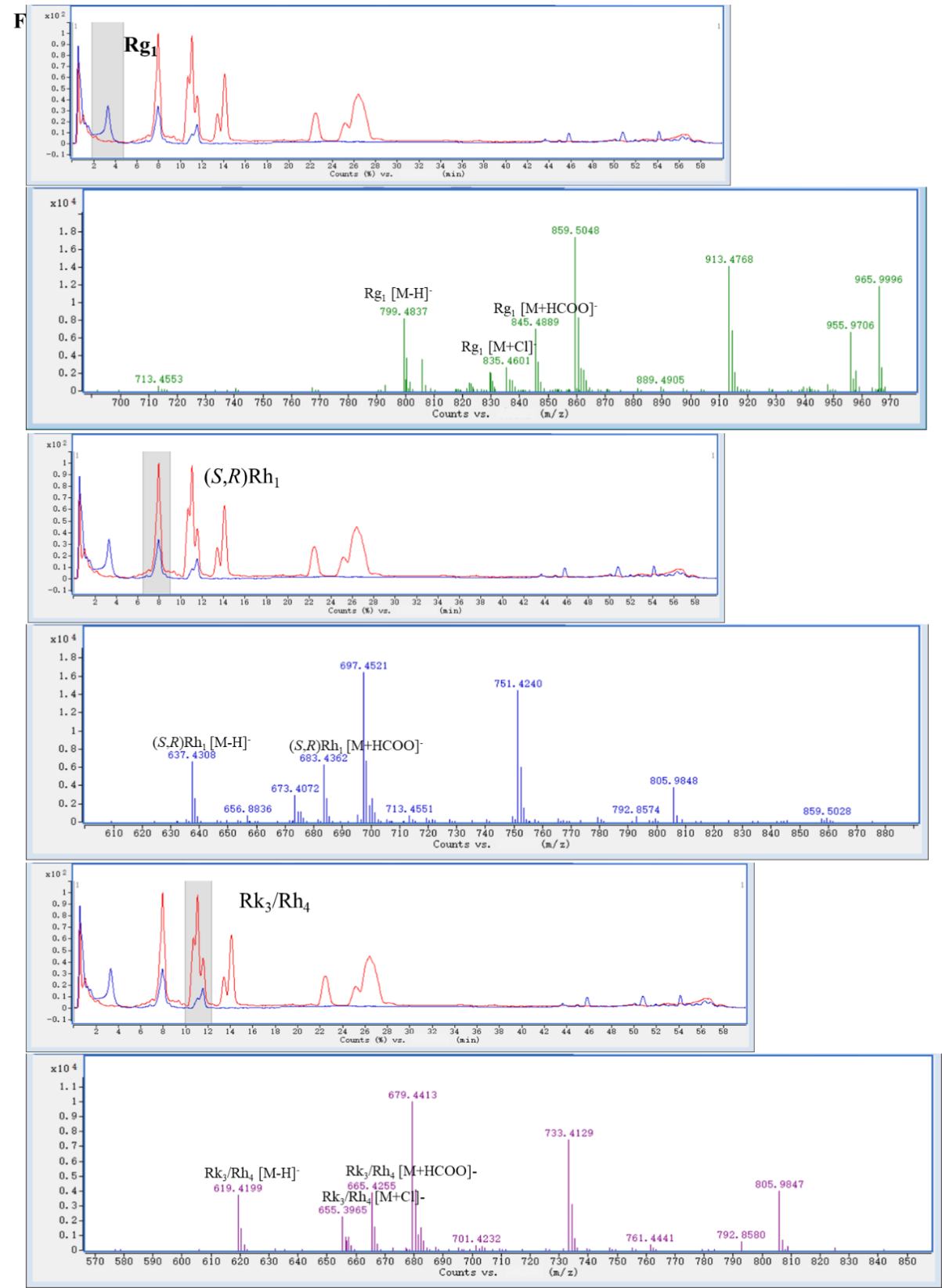


C


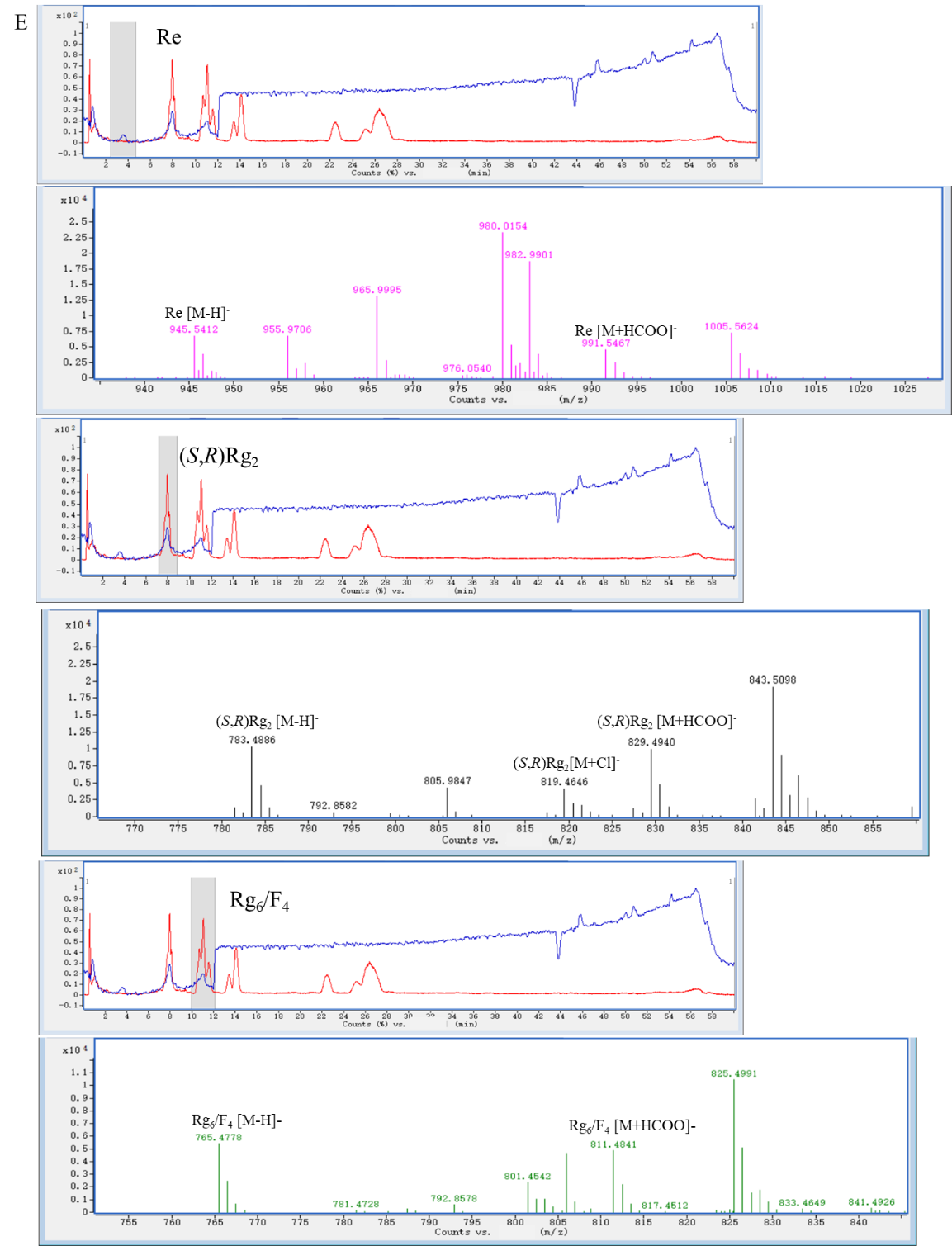


D


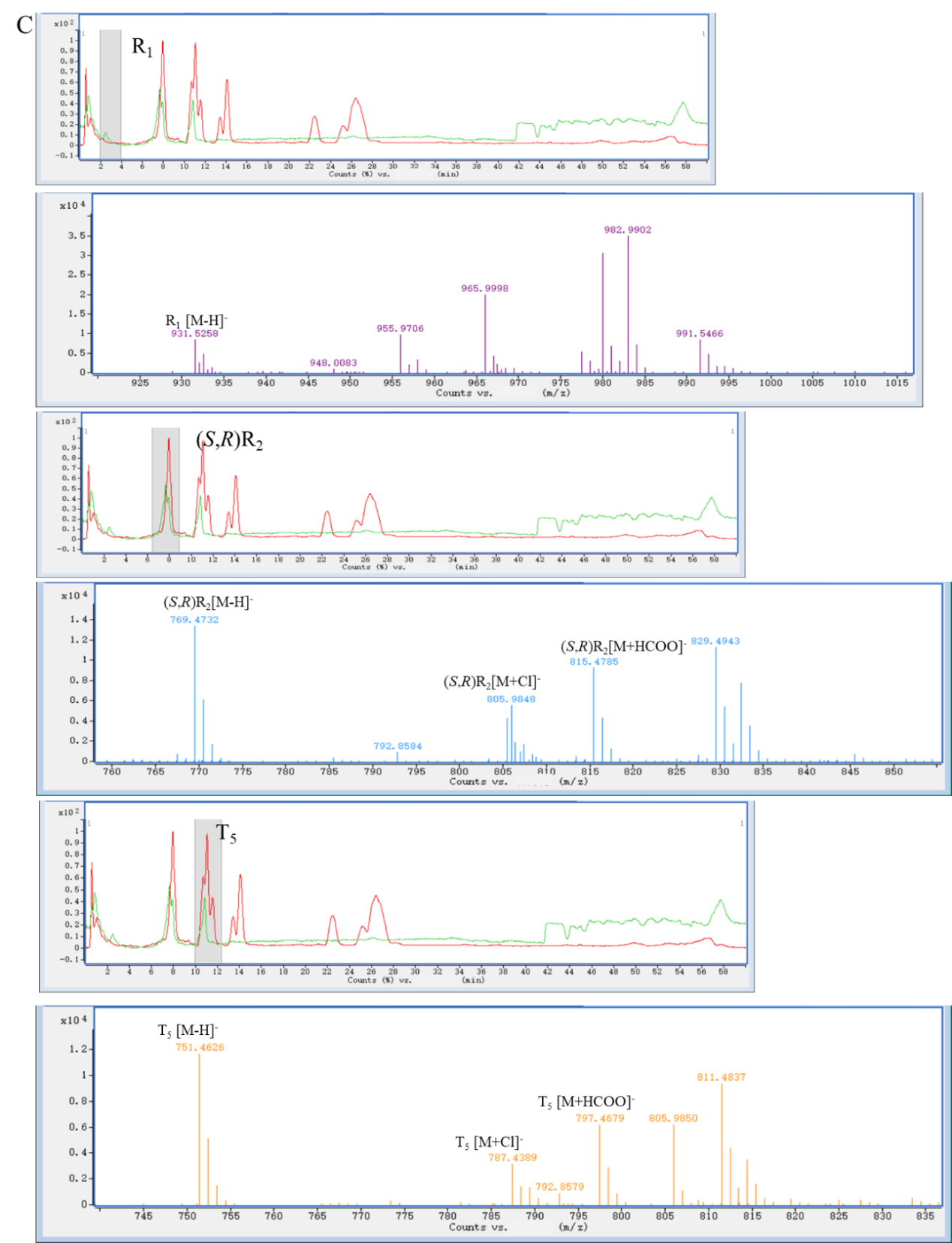


E

**S1 Fig.** LC-MS analysis of transformation products of ginsenosides Rb_1_, Rd, Rg_1_, Re, notoginsenoside R_1_ by *A. tubingensis*.


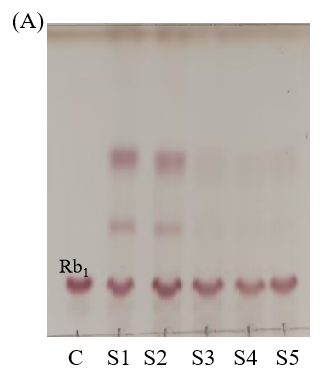

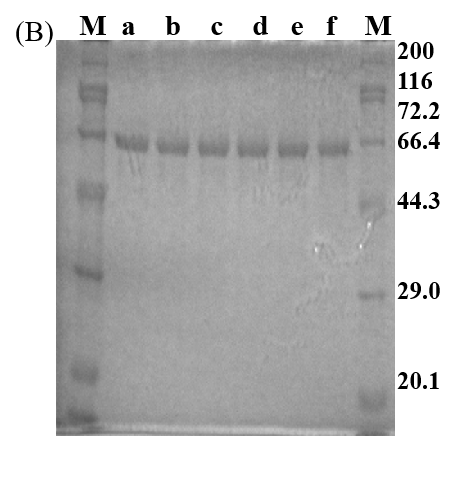


**S2 Fig.** Enzyme characterization of *β-*ginsenosidase. (A) TLC analysis of ginsenosides Rb_1_ transformed by enzyme of different fractions. C: authentic ginsenosides; S1-S2: the fraction enzyme of ginsenoside-transformation activity; S3-5: the other fraction enzyme. (B) SDS-PAGE analysis of the purified *β*-glucosidase from *A. tubingensis* after protein staining with Coomassie Brilliant Blue solution. M: protein marker, a-f: purified enzyme.

**
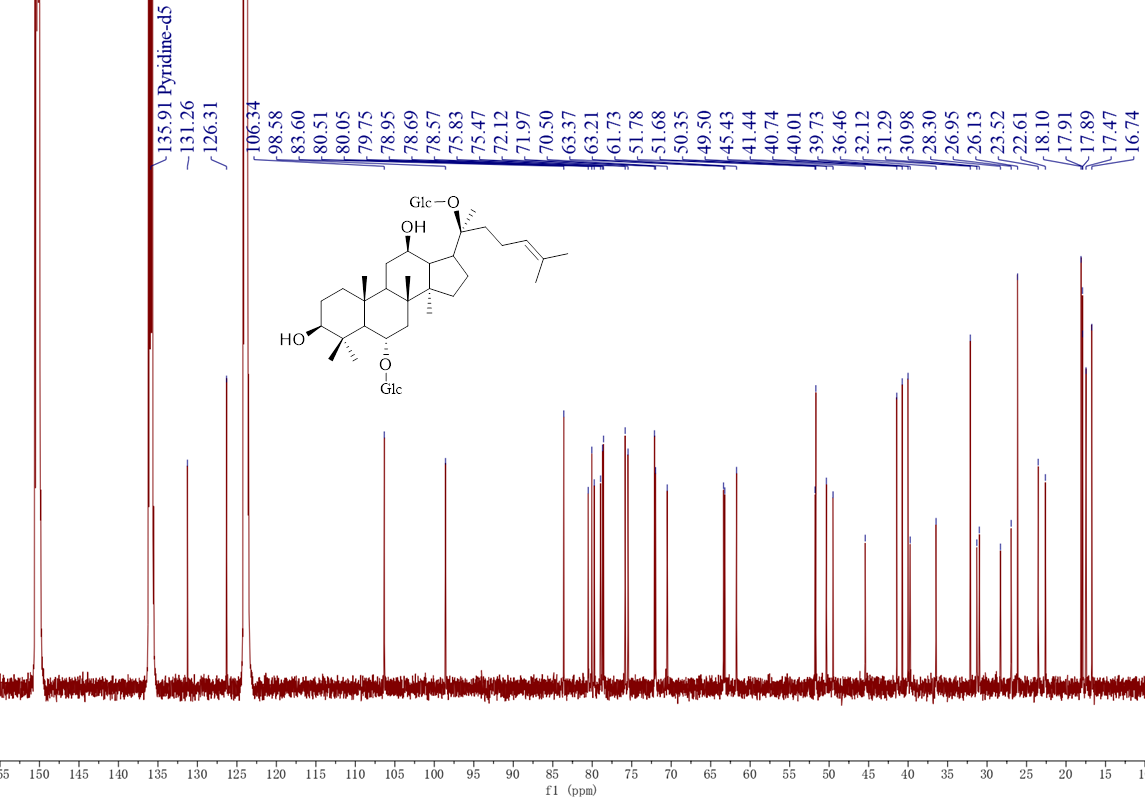
**

**S3 Fig.** ^13^C spectrum (150MHz, C_5_D_5_N) of **1**

**
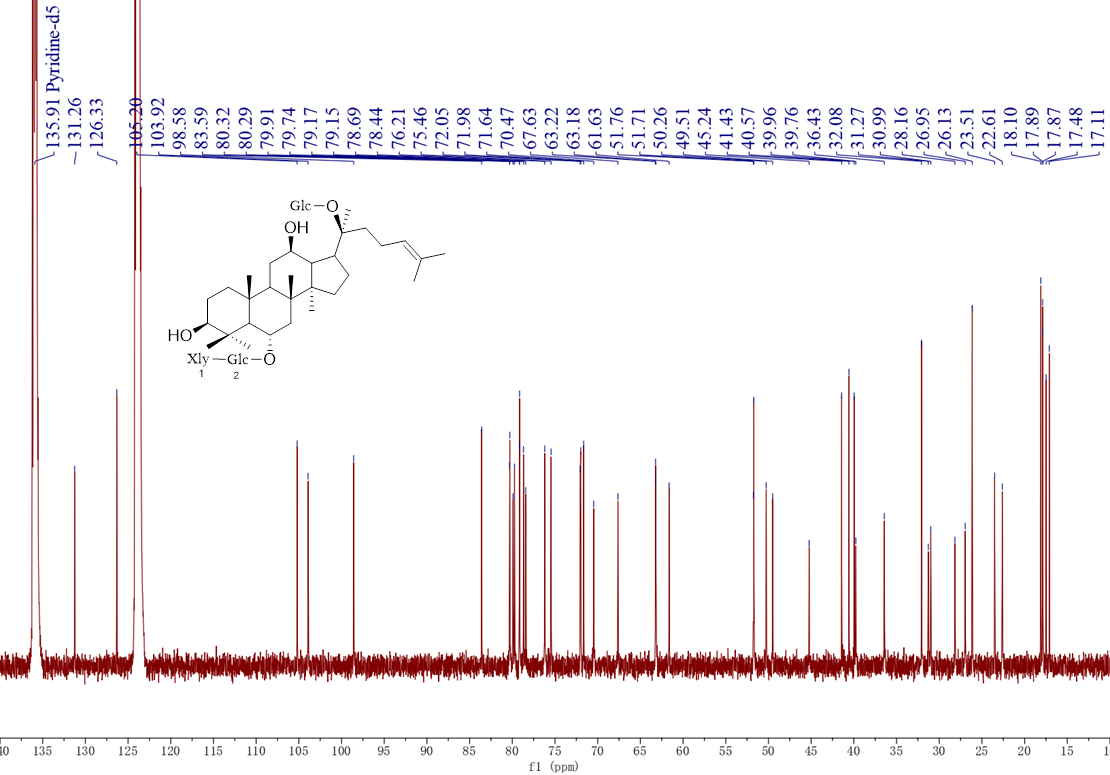
**

**S4 Fig.** ^13^C spectrum (150MHz, C_5_D_5_N) of **2**

**
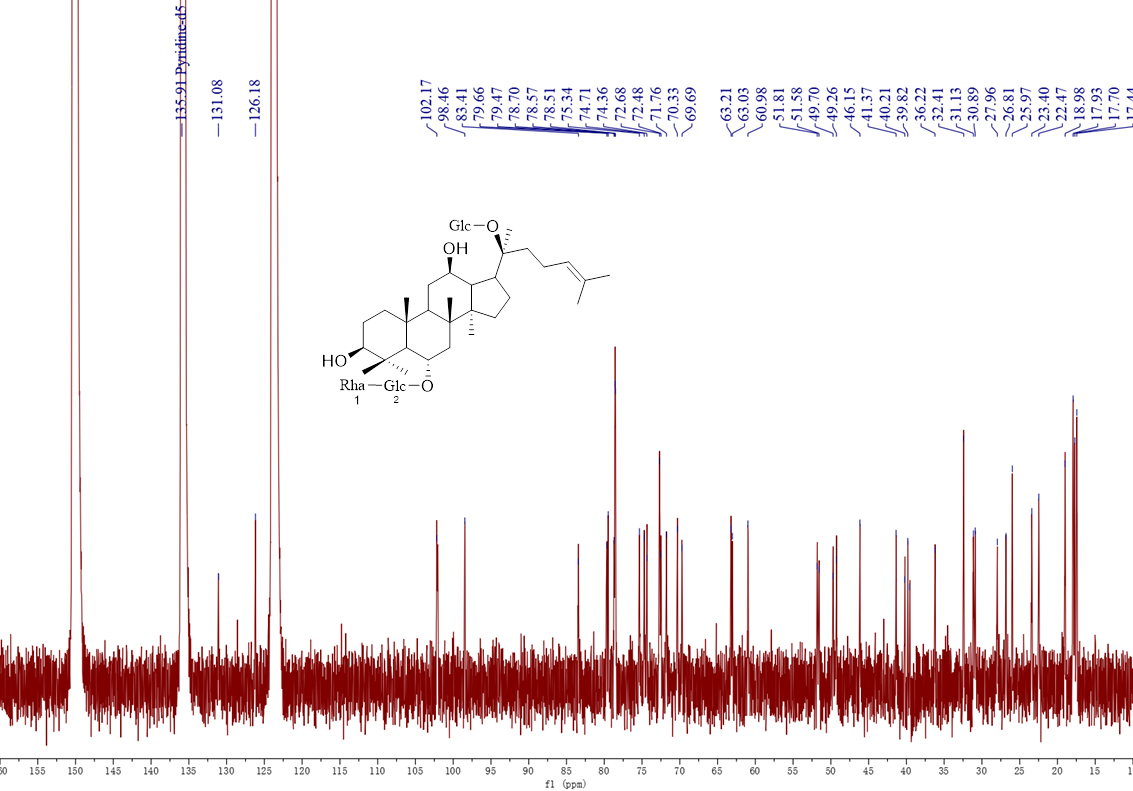
**

**S5 Fig.** ^13^C spectrum (150MHz, C_5_D_5_N) of **3**

**
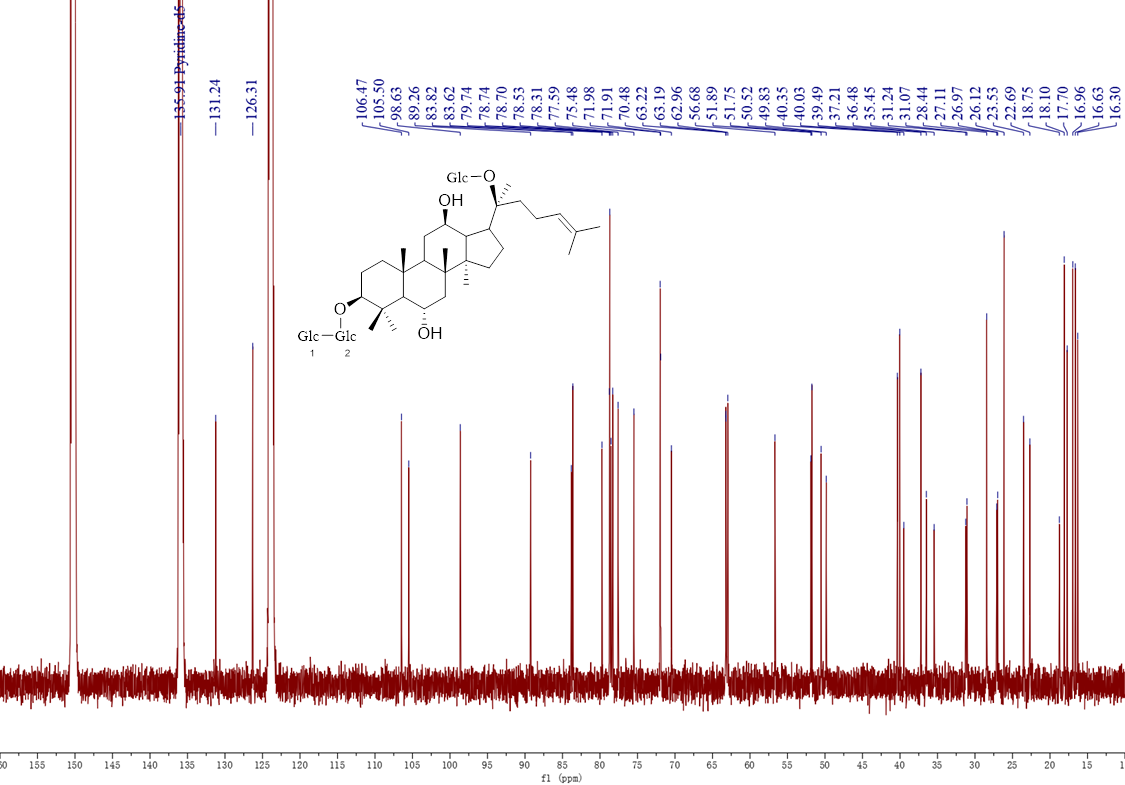
**

**S6 Fig.** ^13^C spectrum (150MHz, C_5_D_5_N) of **4**


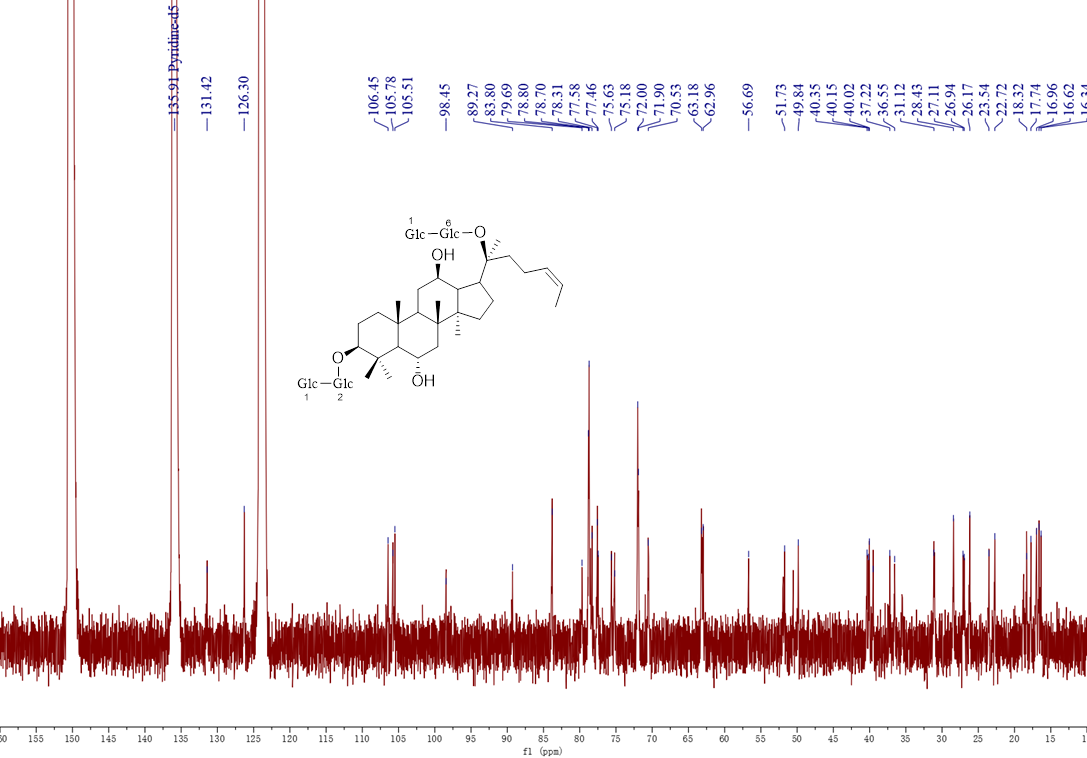


**S7 Fig.** ^13^C spectrum (150MHz, C_5_D_5_N) of **5**

**S1Table** ^13^C NMR data for compounds **1**–**5** in C_5_D_5_N.

| Carbon No. | | **1** | **2** | **3** | Carbon No. | **4** | **5** | Carbon No. | **5** |
| --- | --- | --- | --- | --- | --- | --- | --- | --- | --- |
| 1 | 39.7 | | 39.8 | 40.2 | 1 | 39.5 | 40.0 | Glc |  |
| 2 | 28.3 | | 28.2 | 28.0 | 2 | 27.0 | 26.9 | 1```` | 105.8 |
| 3 | 78.9 | | 79.2 | 78.4 | 3 | 89.3 | 89.3 | 2```` | 75.6 |
| 4 | 40.7 | | 40.6 | 39.8 | 4 | 40.0 | 40.2 | 3```` | 71.9 |
| 5 | 61.7 | | 61.6 | 61.0 | 5 | 56.7 | 56.3 | 4```` | 77.6 |
| 6 | 80.5 | | 80.3 | 74.7 | 6 | 18.7 | 18.3 | 5````  6 | 78.3 |
| 7 | 45.4 | | 45.2 | 46.2 | 7 | 35.4 | 35.4 | 6```` | 63.2 |
| 8 | 41.4 | | 41.4 | 41.4 | 8 | 40.3 | 40.4 |  |  |
| 9 | 50.3 | | 50.3 | 49.7 | 9 | 50.5 | 50.0 |  |  |
| 10 | 40.0 | | 40.0 | 39.6 | 10 | 37.2 | 37.2 |  |  |
| 11 | 31.3 | | 31.0 | 30.0 | 11 | 31.1 | 31.1 |  |  |
| 12 | 70.5 | | 72.0 | 70.3 | 12 | 70.5 | 70.2 |  |  |
| 13 | 49.5 | | 49.5 | 49.3 | 13 | 49.8 | 49.6 |  |  |
| 14 | 51.7 | | 51.8 | 51.6 | 14 | 51.8 | 51.7 |  |  |
| 15 | 31.0 | | 31.3 | 31.1 | 15 | 31.2 | 31.1 |  |  |
| 16 | 26.9 | | 27.0 | 26.8 | 16 | 27.1 | 27.1 |  |  |
| 17 | 51.8 | | 51.7 | 51.8 | 17 | 51.9 | 51.9 |  |  |
| 18 | 17.9 | | 17.9 | 17.1 | 18 | 16.3 | 16.6 |  |  |
| 19 | 17.9 | | 17.9 | 17.4 | 19 | 16.6 | 17.0 |  |  |
| 20 | 83.6 | | 83.6 | 83.4 | 20 | 83.8 | 83.8 |  |  |
| 21 | 22.6 | | 22.6 | 22.5 | 21 | 22.7 | 22.7 |  |  |
| 22 | 36.5 | | 36.4 | 36.2 | 22 | 36.5 | 36.5 |  |  |
| 23 | 23.5 | | 23.5 | 23.4 | 23 | 23.5 | 23.5 |  |  |
| 24 | 126.3 | | 126.3 | 126.2 | 24 | 126.3 | 126.3 |  |  |
| 25 | 131.3 | | 131.3 | 131.1 | 25 | 131.2 | 131.4 |  |  |
| 26 | 26.1 | | 26.1 | 26.0 | 26 | 26.1 | 26.2 |  |  |
| 27 | 18.1 | | 17.1 | 17.9 | 27 | 18.1 | 17.9 |  |  |
| 28 | 32.1 | | 32.1 | 32.4 | 28 | 28.4 | 28.4 |  |  |
| 29 | 16.7 | | 18.1 | 17.7 | 29 | 17.0 | 17.0 |  |  |
| 30 | 17.5 | | 17.5 | 17.1 | 30 | 17.7 | 17.7 |  |  |
| 6-O-Glc |  | |  |  | 3-O-Glc |  |  |  |  |
| 1` | 106.3 | | 103.9 | 101.7 | 1` | 105.5 | 105.5 |  |  |
| 2` | 75.8 | | 80.3 | 78.7 | 2` | 83.6 | 83.8 |  |  |
| 3` | 80.1 | | 78.4 | 79.7 | 3` | 78.5 | 79.7 |  |  |
| 4` | 72.1 | | 70.5 | 72.7 | 4` | 71.7 | 72.0 |  |  |
| 5` | 78.7 | | 79.9 | 78.6 | 5` | 78.7 | 78.8 |  |  |
| 6` | 63.4 | | 63.2 | 63.2 | 6` | 63.2 | 63.2 |  |  |
| Xyl(or Rha) |  | |  |  | Glc |  |  |  |  |
| 1`` |  | | 105.2 | 102.2 | 1`` | 106.5 | 106.5 |  |  |
| 2`` |  | | 75.5 | 72.5 | 2`` | 77.6 | 77.5 |  |  |
| 3`` |  | | 79.2 | 71.8 | 3`` | 78.3 | 78.8 |  |  |
| 4`` |  | | 71.6 | 74.4 | 4`` | 72.0 | 72.0 |  |  |
| 5``  6 |  | | 67.6 | 69.7 | 5``  6 | 78.3 | 78.7 |  |  |
| 6`` |  | |  | 19.0 | 6`` | 63.2 | 63.0 |  |  |
| 20-O-Glc |  | |  |  | 20-O-Glc |  |  |  |  |
| 1``` | 98.6 | | 98.6 | 98.5 | 1``` | 98.6 | 98.5 |  |  |
| 2``` | 75.5 | | 76.2 | 75.3 | 2``` | 75.5 | 75.1 |  |  |
| 3``` | 79.7 | | 79.7 | 79.5 | 3``` | 79.7 | 78.7 |  |  |
| 4``` | 72.0 | | 72.0 | 79.0 | 4``` | 71.9 | 71.9 |  |  |
| 5``` | 78.6 | | 78.7 | 78.5 | 5``` | 78.7 | 77.3 |  |  |
| 6``` | 63.2 | | 63.2 | 63.0 | 6``` | 63.0 | 70.5 |  |  |
