# Supplementary figures and images for: The transformation pathways and optimization of conditions for preparation minor ginsenosides from Panax notoginseng root by the fungus Aspergillus tubingensis
Source: PLoS One. 2025 Mar 3;20(3):e0316279. doi: 10.1371/journal.pone.0316279 (PMC11875379; doi:10.1371/journal.pone.0316279)

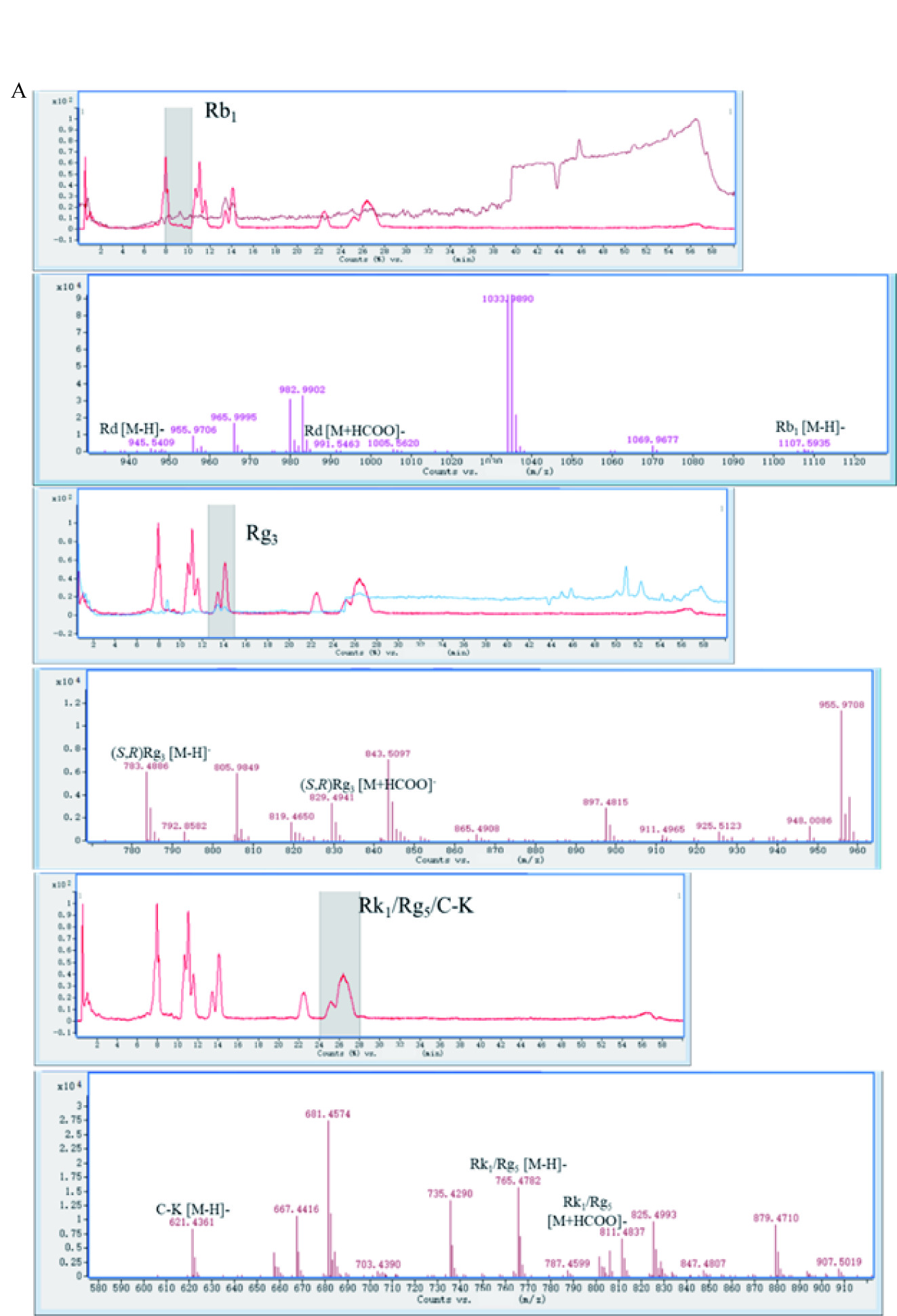

Supplement: S1 Fig — (TIFF) [file pone.0316279.s002.tif]

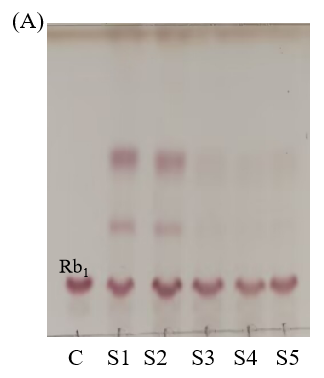

Supplement: S2 Fig — (TIFF) [file pone.0316279.s003.tif]

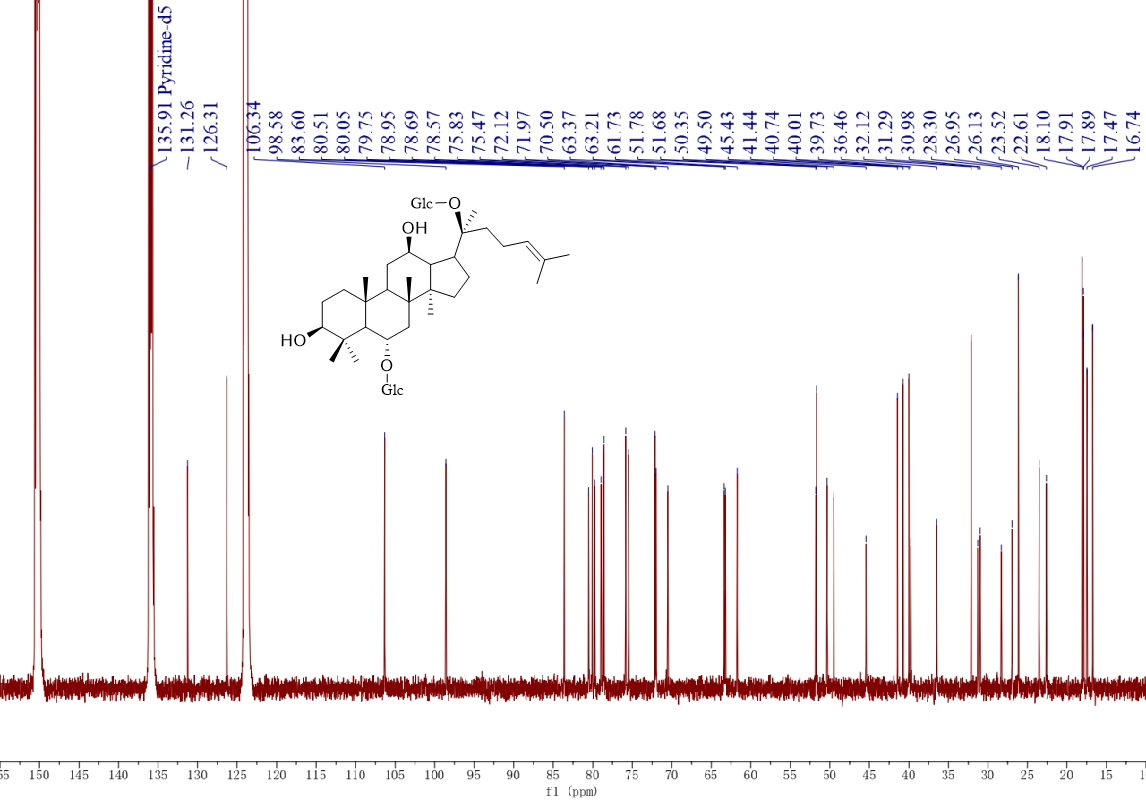

Supplement: S3 Fig — (TIF) [file pone.0316279.s004.tif]

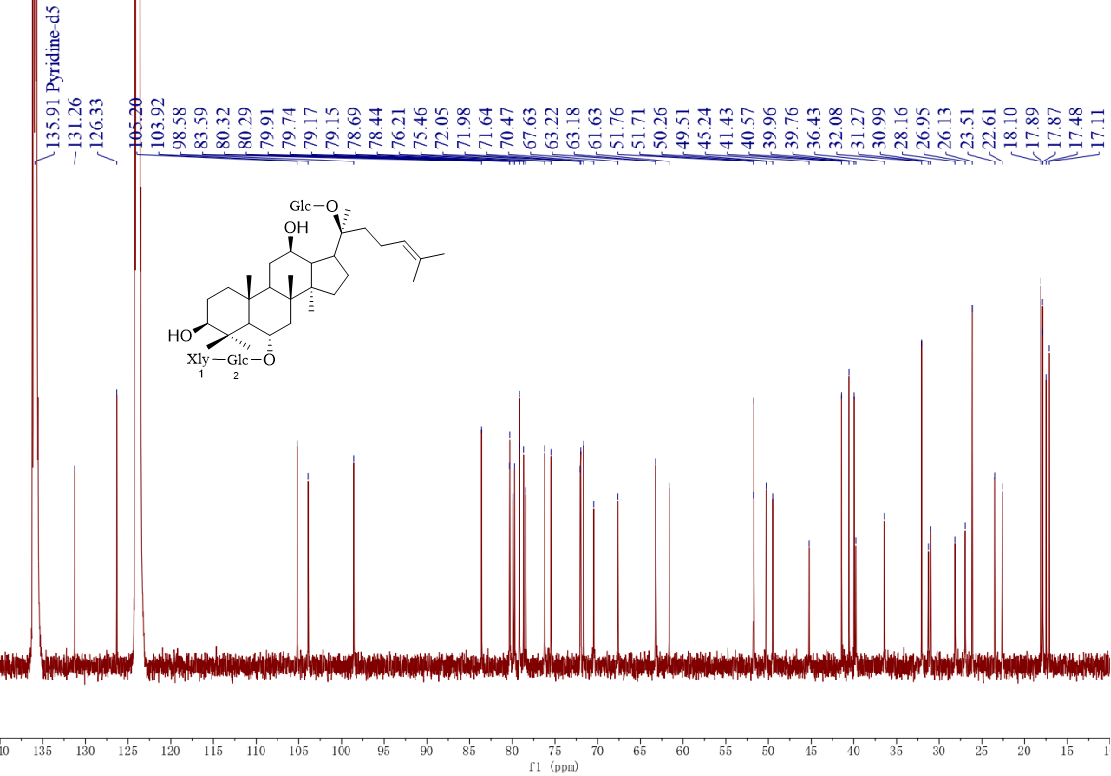

Supplement: S4 Fig — (TIF) [file pone.0316279.s005.tif]

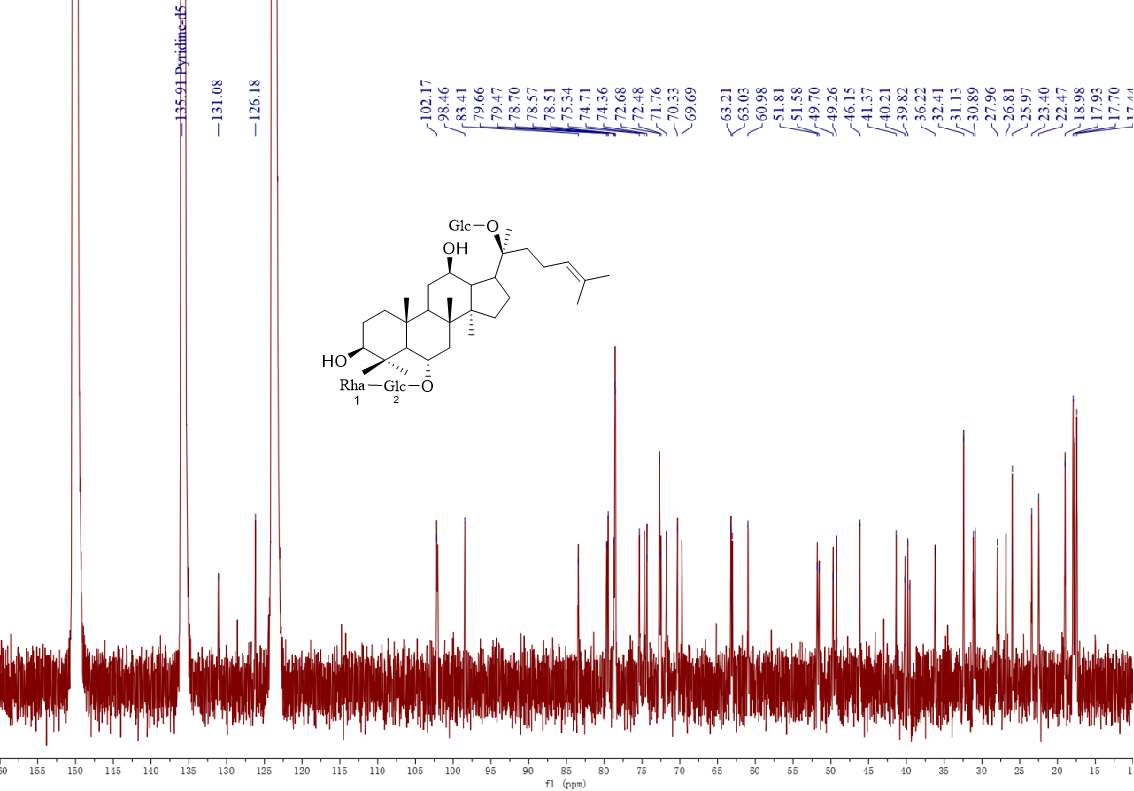

Supplement: S5 Fig — (TIF) [file pone.0316279.s006.tif]

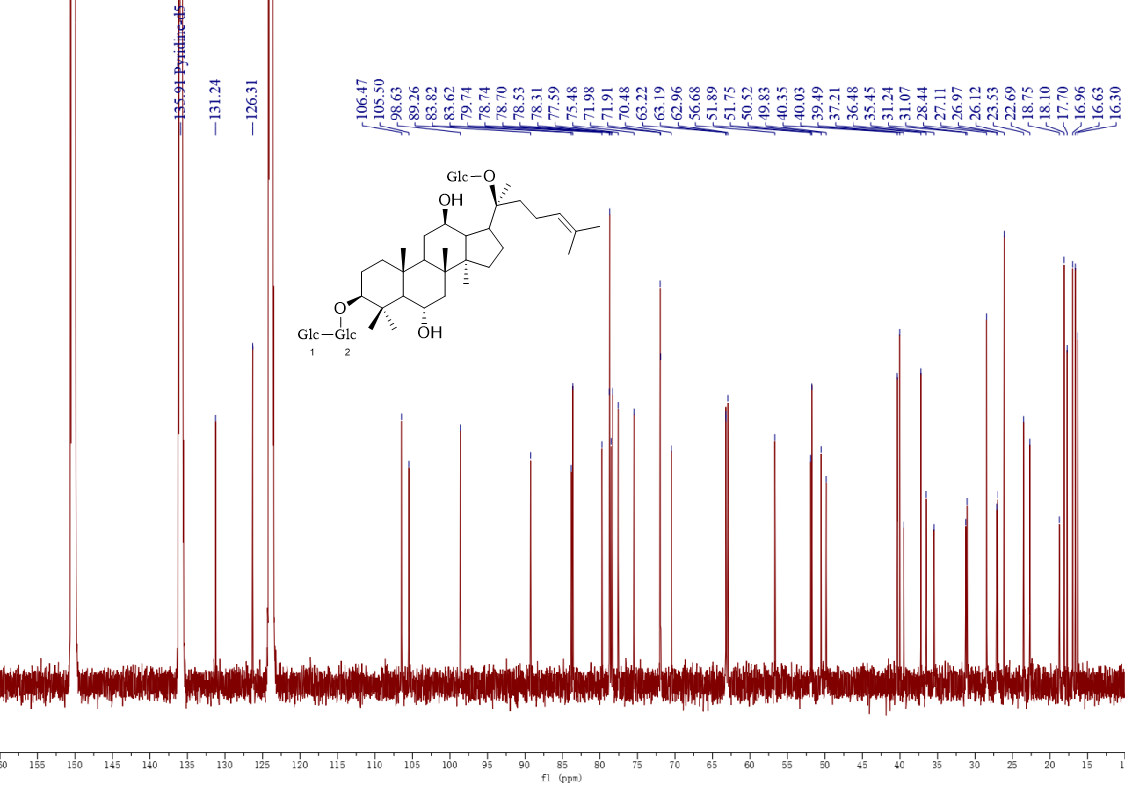

Supplement: S6 Fig — (TIF) [file pone.0316279.s007.tif]

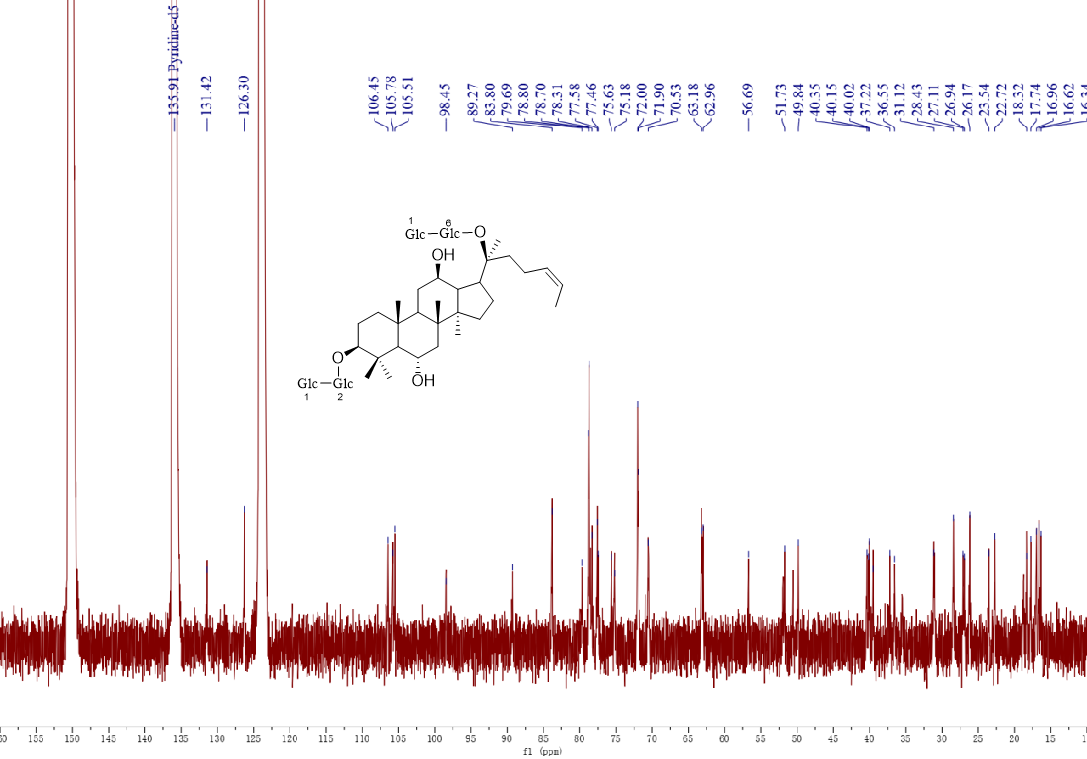

Supplement: S7 Fig — (TIF) [file pone.0316279.s008.tif]
